# A cautionary tale of batch corrections on confounded microbiome community profiles

**DOI:** 10.1002/imo2.70025

**Published:** 2025-05-19

**Authors:** Alicia J. Foxx, Adam R. Rivers

**Affiliations:** ^1^ Department of Plant Biology and Conservation Northwestern University Evanston Illinois USA; ^2^ United States Department of Agriculture, Agricultural Research Service Genomics and Bioinformatics Research Unit Gainesville Florida USA; ^3^ Present address: The Chicago Botanic Garden Glencoe Illinois USA; ^4^ Present address: Northwestern University Evanston Illinois USA

## Abstract

We use a case study of seed microbiomes to assess the performance of five batch effects correction algorithms (BECAs) (zero‐mean centering (ZMC), Ratio‐A, conditional quantile regression (ConQuR), partial least squares discriminant analysis (PLSDA), and weighted PLSDA on a confounded data set in which the covariate of interest (plant species) does not appear in all batches (studies). We show incomplete batch effects removal in all cases, which calls for careful application of and future work on BECAs.

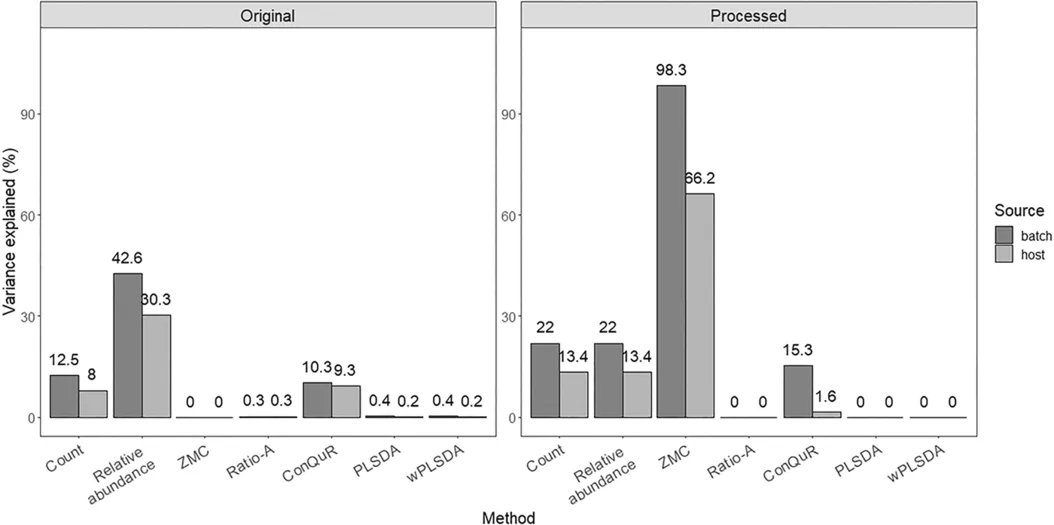

Variation from batch effects in microbiome research harms reproducibility and generalizability [1] and is a hit to reliability that microbiome research cannot afford [2]. Batch effects are an issue in individual studies, such as when data is collected over space, time, or on separate cohorts. Batch effects are exacerbated in research that synthesizes microbiome sequence data across multiple studies, as they often lack fully or partially overlapping study species. Batches can account for more variation in microbiome community profiles than covariates of interest (e.g., phenotypes [3]), and ultimately, jeopardize the use of synthesized microbiome findings in research areas such as agriculture and clinical research, which rely on this information for making recommendations, e.g., [4].

There are limits to the mitigation of batch effects from standardizing protocols and analysis pipelines across studies [5]. Therefore, batch effects correction algorithms (BECAs) are crucial tools developed to remove the influence of batches on a data set. Another key issue in microbiome secondary analyses is when batches and covariates of interest are confounded, resulting in covariates of interest that do not appear in all batches [6, 7]. Confounded designs for batch effects, such as when a species appears in some, but not all studies (or batches) [8, 9], are critically overlooked in microbiome syntheses. The use of BECAs on confounded datasets results in some or all of the batch effects remaining following corrections [7], and an increase in statistical false positives or negatives in downstream analyses, and reduces the accuracy of machine learning tasks [10]. For example, Sonesone et al. [8] found greater bias and lower prediction accuracy in machine learning models when using confounded gene expression data for the classification of cancer status. Furthermore, confounded designs are common in meta‐analyses and integrative or secondary analyses that reanalyze sequence data of nonhuman host organisms (e.g., Figure 1A) and require a deeper examination.

**FIGURE 1:**
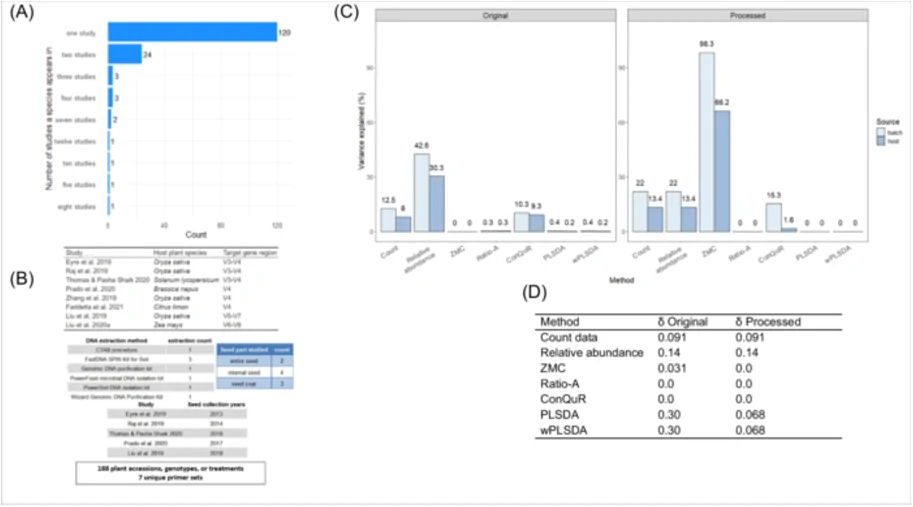
Outlook of variability of underlying studies and BECA outcomes. (A) The count of seed microbiome studies that a species appears in. Seed microbiomes have been evaluated on 157 unique plant species, and most (120) of them appear in only one study, demonstrating the extremely confounded nature of seed microbiome studies typical of nonhuman microbiome research (based on Web of Science search details in Figure S1). (B) Table of study name, host species, target gene region, and seed processing details. (C) Redundancy analysis outcomes of percent variance explained for wanted (host) and unwanted variables (study batch) variables across three data set inputs. Processed data includes center log‐ratio transformation and zero‐imputation. (D) Guided Principal Component Analysis results denoting the magnitude of batch effects on the datasets [11]. Guided PCA performs a residual PCA to remove variation due to known batch variables in the data set, then quantifies the proportion of variation due to batch effects from the first principal components of the residual PCA using the test statistic, delta (δ), which quantifies the amount of variation due to batch effects in genomic data [11]. Large δ values from this test indicate larger batch effects [11]. Larger delta values (δ) indicate larger batch effects. All δ values were significant at *p* < 0.001.

## 1 EXAMINING BATCH EFFECTS ON SEED MICROBIOME COMMUNITY PROFILES

We used seed microbiomes as a case study and evaluated the performance of five BECAs on seed microbiome community profiles on 345 samples across eight studies (Table S1, Figure 1B). This synthesis is on a confounded data set, in which all plant species do not occur in each batch. We compared the performance of BECAs developed specifically to address batch corrections of amplicon data and implemented with R: zero‐mean centering, Ratio‐A, conditional quantile regression (ConQuR) [3], partial least squares discriminant analysis batch correction (PLSDA‐batch), and weighted PLSDA‐batch (wPLSDA‐batch) developed for imbalanced batches and covariates (Figure S2, Methods S1, [7]). We assessed the variance explained following application of each BECA for the unwanted batches and whether they maintained explanatory ability on the host species using a redundancy analysis (RDA). RDA simultaneously estimates the variance explained by multiple variables (e.g., batch and covariate of interest) in multivariate datasets. The RDA demonstrated that BECAs differed in how much variation the host plant and batch explained for the datasets. For raw datasets that were not processed with center‐log ratio transformed, relative abundance explained the most variation in the wanted (host; 30.3%) and unwanted variables (study; 42.6%), whereas ZMC explained 0% variation in either variable (Figure 1C). Additionally, PLSDA, wPLSDA, & Ratio‐A explained between 0% and 0.4% of the variation for both variables with and without processing (Figure 1C). On the other hand, the ZMC processed data set explained the highest amount of variation in the wanted (66.2%) and unwanted variables (98.3%).

Ultimately, several BECAs helped to reduce the magnitude of batch effects but correspondingly had varying influences on how well the BECA reduced variance explained by the batch variable (study) (Figure 1C,D). Generally, no method reduced variation explained by batch to zero while simultaneously maintaining the variance explained by the host plant variable. Weighted PLSDA‐batch did not reduce batch effects for these imbalanced data sets, contrary to our expectations (Figure 1C). Interestingly, CLR and zero imputation processing with some BECAs resulted in the highest amount of variation explained in the wanted variable, and some BECAs resulted in indetectable batch severity. Some BECAs helped remediate batch effects in batch‐covariate imbalanced datasets.

## 2 IMPLICATIONS OF BATCH‐COVARIATE IMBALANCE

The future of microbiome science requires the development of approaches to robustly address batch‐covariate confounded data, which is a widespread problem in microbiome syntheses. Here, we purposefully violated one requirement of many BECAs and used a confounded data set, which limited batch removal success. What is the recourse for synthesizing microbiome data with severe batch‐covariate imbalance since confounded structures are common in nonhuman synthesis? Currently, modeling approaches such as univariate mixed effects models and meta‐analysis models are the best approach. These approaches account for, rather than remove unwanted variation [10, 12]. Mixed effects models incorporate batch as a random effect which controls for nonindependence between batches by constraining the covariate levels to have the same intercept, slope, or intercept and slope. Specifically, mixed effects models estimate and partition unwanted variation from batches as random effects and control for nonindependence due to samples arising from the same batch. Unfortunately, linear and generalized linear models have the drawback of information loss while reducing multivariate data to univariate means instead of incorporating multivariate, multi‐feature nature of microbiome profiles, for example. Mixed effects models and mixed effects meta‐analysis models would help to explicitly model batch in imbalanced batch‐covariate data and can consider interactions between batch and class variables, which most BECAs do not address (but see [7]). Without BECAs appropriate for confounded datasets, researchers should also consider excluding studies to infer comprehensively about a more focused subset of studies (e.g., excluding studies that do not include both men and women in a fecal microbiome study) and employ BECAs.

## 3 CALL FOR FUTURE WORK TO ADDRESS BATCHES IN CONFOUNDED DATASETS

Some BECAs have been specifically developed for microbiome community profiles that require case‐control pairings, which were not able to be applied in these BECA evaluations [5, 13]. This is because the underlying seed microbiome studies lacked control groups and often had the goal of characterizing the core microbiome without treatment interventions (see [14])—like many other microbiome studies. However, it is possible that case‐control‐reliant BECAs may help mitigate batch effects for batch‐covariate confounded data due to the standardization of the batches relative to a reference. One potential avenue is to have a common thread across studies is to include individuals from the same source for benchmarking, such as model species like *Arabidopsis thaliana, Oryza sativa,* or *Brachypodium distachyon* for plant microbiome studies [15]. This approach can be used to normalize target species to the standard for comparisons across studies and help with a shortcoming of our analysis: we lack the ground truth of the data without batch effects with which to compare. Generally, there is a critical need for continued development of BECAs and other multivariate methods to account for unwanted variation in realistic cases in which batch and class are imbalanced as there continue to be mountains of publicly available data ripe for re‐evaluating for drawing new conclusions and testing generalizations; this will further BECA development will aid microbiome advancement.

## AUTHOR CONTRIBUTIONS

**Alicia J. Foxx**: Conceptualization; investigation; methodology; validation; writing—original draft; writing—review and editing; formal analysis; project administration. **Adam R. Rivers**: Resources; writing—review and editing.

## CONFLICT OF INTEREST STATEMENT

The authors declare no conflicts of interest.

## ETHICS STATEMENT

No animals or humans were involved in this study.

## Supporting information


**Figure S1.** Web of Science Search results for studies that quantified the seed microbiome.
**Figure S2.** Approaches used to generate and process datasets with CLR and zero impute derived from raw count feature matrix with batches.
**Methods S1.** Microbiome data processing and bioinformatic pipeline processing.
**Table S1.** Table of study name, gene region, SRA project number, and study DOI.

## Data Availability

The data that support the findings of this study are openly available in Mendeley data at https://doi.org/10.17632/5xrfg5dym6.1. Accession information for publicly available data is listed in Table S1 and code to reproduce these analyses is available at https://doi.org/10.17632/5xrfg5dym6.1. Supplementary materials (figures, tables, and graphical abstract may be found in the online DOI or iMeta Science http://www.imeta.science/imetaomics/.
