# Supplementary material for: A cautionary tale of batch corrections on confounded microbiome community profiles
Source: IMetaOmics. 2025 May 19;2(3):e70025. doi: 10.1002/imo2.70025 (PMC12806050; doi:10.1002/imo2.70025)
Supplement: Supplementary file 1 — Figure S1. Web of Science Search results for studies that quantified the seed microbiome. Figure S2. Approaches used to generate and process datasets with CLR and zero impute derived from raw count feature matrix with batches. Methods S1. Microbiome data processing and bioinformatic pipeline processing. Table S1. Table of study name, gene region, SRA project number, and study DOI. [file IMO2-2-e70025-s001.docx]

**Supporting Information to:** A cautionary tale of batch corrections on confounded microbiome community profiles

**Running Title:** benchmarking batch correction methods

**Authors:** Alicia J. Foxx^1,2^*, Adam R. Rivers^3^

^1^Negaunee Institute for Plant Conservation Science and Action, The Chicago Botanic Garden, 1000 Lake Cook Road, Glencoe, IL, 60022, USA

^2^Department of Plant Biology and Conservation, Northwestern University, 2205 Tech Drive, Evanston, IL, 60201, USA

^3^United States Department of Agriculture, Agricultural Research Service, Genomics and Bioinformatics Research Unit, 1600 SW 23rd Drive Gainesville, FL 32608, USA

*Corresponding author: E: [afoxx@chicagobotanic.org](mailto:alicia.foxx@usda.gov)

**Table S1.** Table of study name, gene region, SRA project number, and study DOI.

| **Study** | **No. of samples** | **Study accession information** | **Study DOI** |
| --- | --- | --- | --- |
| Eyre et al. 2019 | 24 | PRJNA531035 | 10.1094/PBIOMES-01-19-0009-R |
| Raj et al. 2019 | 56 | PRJNA529046 | 10.1186/s12864-019-6334-5 |
| Thomas & Pasha Shaik 2020 | 2 | PRJNA552773 | 10.1007/s00248-019-01440-5 |
| Prado et al. 2020 | 198 | PRJEB31847 | 10.1038/s41598-020-60591-5 |
| Zhang et al. 2019 | 24 | PRJNA361068 | 10.1007/s00344-018-9812-0 |
| Faddetta et al. 2021 | 10 | PRJEB34309 | 10.1038/s41598-021-86399-5 |
| Liu et al. 2019 | 24 | PRJNA530737 | 10.1007/s10725-018-0467-4 |
| Liu et al. 2020 | 27 | PRJNA510484 | 10.1007/s13205-019-2034-8 |


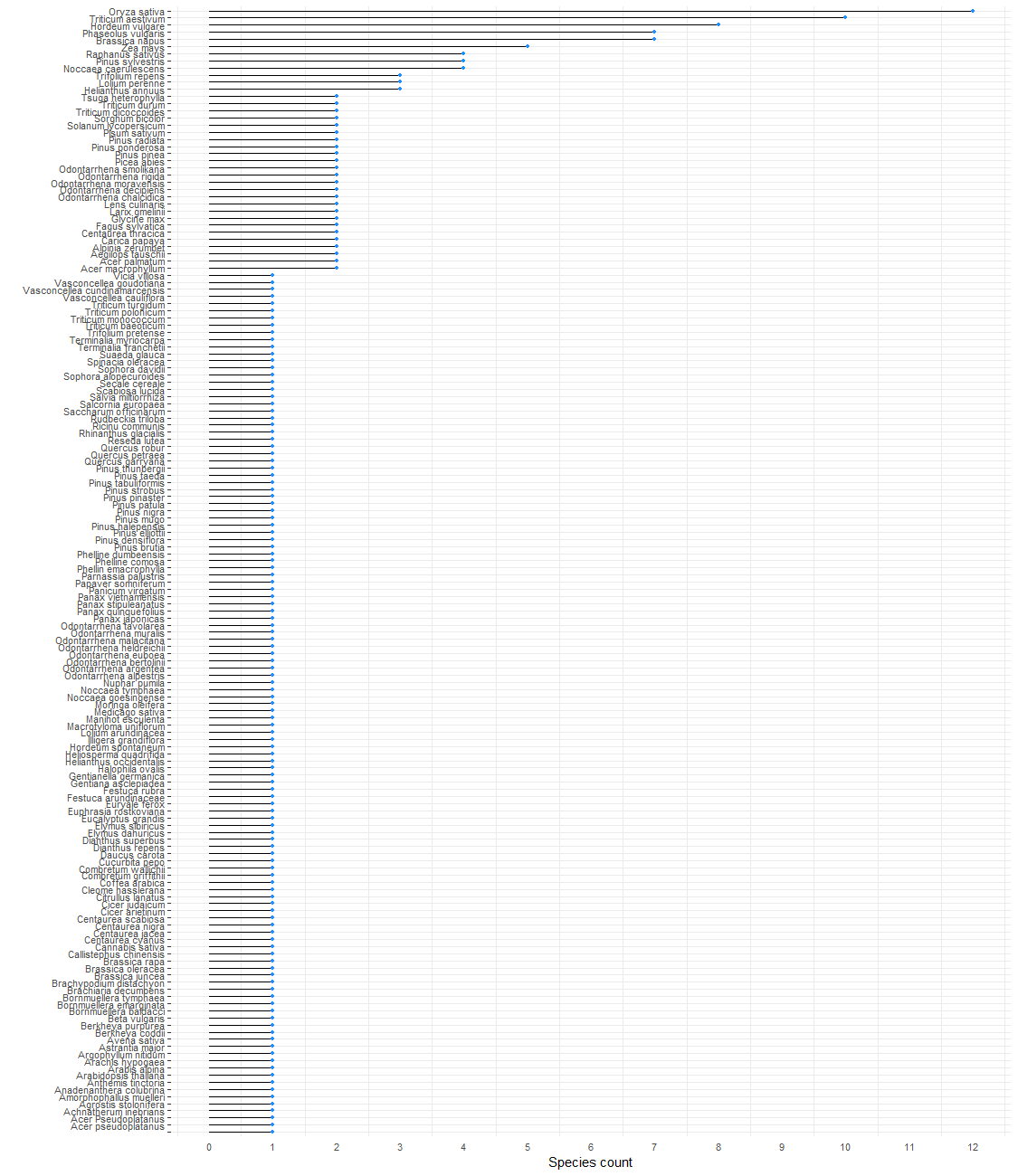


**Figure S1**. Web of Science Search results for studies that quantified the seed microbiome. This search was conducted on 2023-11-21 using the following search terms topic: [seed*] AND [microbi* OR Endophyt* OR Epiphyt* OR microbiota OR bacteriome OR mycobiome] AND [Metagenom* OR High throughput OR shotgun OR metabarcod* OR amplicon]. This resulted in 157 unique plant species in seed microbiome studies. Most species only appear in one study and a few species appear in multiple studies (all agronomic).


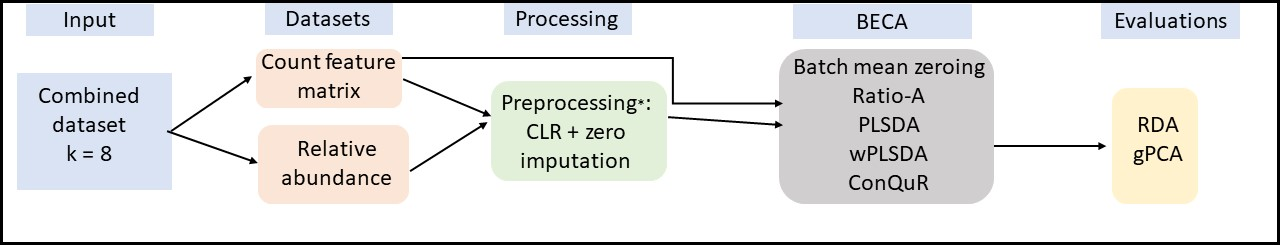


**Figure S2.** Approaches used to generate and process datasets with CLR and zero impute derived from raw count feature matrix with batches. *Denotes variability in whether zero imputation and CLR preceded or followed the correction, which differed for some BECA due to issues of negative values (see SI Methods).

**Methods S1.** Microbiome data processing and bioinformatic pipeline processing.

*Seed microbiome & studies*

We used eight studies that assessed the seed microbiomes of five agronomic plant species to evaluate BECA performance (Figure S1). The methods of seed handling and processing vary widely for seed microbiome studies, which influences microbiome diversity and composition [1].

*Microbiome data processing*

We downloaded publicly available FASTQ sequence files (Table S1) for each study using a standard bioinformatic pipeline (Methods S1: Microbiome data processing) with Quantitative Insights Into Microbial Ecology version 2 (QIIME 2 v.2020.11[2]. The resulting amplicon sequence variants (ASV) feature table was collapsed at the genus level and further processed and analyzed in R [3] with the *phyloseq* package (Mcmurdie & Holmes, 2013). We filtered out taxa found in less than 0.0005% of the 349 samples resulting in 15,344 ASVs. We also compared processing the data with zero-imputation and center log ratio transformation and performed batch correction tests (Figure S1).

*Batch correction methods*

We compared five BECAs: zero-mean centering approach (ZMC), which adjusts batch means for each ASV to become zero to remove batch variation [4,5]; Ratio-A, which scales all samples by dividing them by the batch-specific arithmetic mean [6,7]; PLSDAbatch uses Partial Least Squares Regression Discriminant Analysis to estimate variation due to the covariate of interest, then deflates the variation due to batch to preserve covariate variation; weighted PLSDAbatch works similarly and corrects feature matrices with imbalanced batch-covariate designs [8]; and Conditional Quantile Regression, ConCuR [9], which is a two-step regression batch correction method that incorporates the zero-inflated and over-dispersed nature of microbiomes (we arbitrarily used the [10] study as the required batch reference with ConQuR).

*Comparative analyses across methods*

We assessed the variance explained following application of each BECA for the unwanted batches and whether they maintained explanatory ability on the host species using a redundancy analysis (RDA) which simultaneously demonstrates the variance explained by multiple variables (e.g., batch and covariate of interest) in multivariate datasets. We also assessed the magnitude of batch effects with a gPCA which detects and estimates the magnitude of batch effects in high-dimensional genomic data using the test statistic, delta (δ) [11]. Large δ values from this test indicate greater amounts of variation due to batch effects [11].

We downloaded publicly available FASTQ sequence files for each study using bash curl commands obtained by searching Sequence Read Archive or European Nucleotide Archive project numbers through SRA explorer [12] (Table S1). We processed each study using a standardized pipeline with Quantitative Insights Into Microbial Ecology version 2 (QIIME 2 v.2020.11[2]). The sequences were in paired-end format and we first demultiplexed the sequence reads and imported them for use in QIIME 2 in the PairedEndFastqManifestPhred33 format. We used DADA2 to denoise, quality-filter, dereplicate and remove chimeras from the sequence data resulting in amplicon sequence variants (ASV) feature tables for each study [13]. ASVs are used to identify sequences with a single nucleotide difference, and this differs from clustering similar operational taxonomic units (OTUs) into OTUs [9]. We aligned the sequences to the Silva database using SILVA_138_99 at 99% sequence similarity using a Naïve Bayes classifier for taxonomic identities. To account for differences in target variable gene region and differences in primer choice, we trained separate classifiers on unique forward and reverse primers for taxonomic classification to extract reads from the reference database targeting the same gene region. The separate feature tables and taxonomies were merged for analyses using the “feature-table merge” command in QIIME 2. We checked the datasets for sequence quality using the online QIIME quality viewer and we chose sequence quality cut-offs for each study at the sequence position below which the quality decreased. We checked the metadata files for appropriate formatting in Keemei [14] for use in QIIME 2 and collapsed ASV feature tables at the genus level.

Merged feature tables were further processed and analyzed in R (v4.2.2) [3]. The QIIME 2 artifacts were converted to phyloseq objects using the “qza_to_phyloseq” function in the “phyloseq” package [15]. We then filtered out taxa found in less than 0.0005% of the 349 samples reducing the number of ASVs from 75,157 to 15,344 ASVs. We filtered ASVs for those classified as bacteria then calculated the relative abundance from the ASV counts. The metadata files were merged in R and correspondingly filtered for samples that mapped to bacteria. We mapped ASV feature tables to batch information (study) and host plant species using sample ID. We processed the data and performed batch corrections using multiple approaches (Figure 1, main text).

We created both a raw count feature matrix and a processed dataset in which we applied CLR and zero imputation to the raw count feature matrix (Figure 1, main text). For processing, we imputed zeros with the "compositions" package in R [16] which determines the detection limit when microbiome counts are rounded zeros and below a count of one [17], then replaces those values with a small positive number that is a fraction of the detection limit (here, 0.67). Following zero imputation, we applied a center-log-ratio transformation (CLR) to the count feature tables to account for data compositionality [18] using the “clr” function in the “compositions” package [16]. Not all BECAs could correct on CLR-transformed and zero imputed data due to negative values, so for zero mean centering (ZMC) we used CLR, then performed ZMC followed by zero imputation. We followed ConQuR batch corrections with zero imputation and CLR as ConQuR requires feature inputs to be counts and not continuous values.

Data and code to reproduce these analyses are available at <https://doi.org/10.17632/5xrfg5dym6.1> [19].

**Supplemental information references**

1. Eyre, Alexander W., Wang, Mengying., Oh, Yeonyee., and Dean, Ralph. A. 2019. “Identification and characterization of the core rice seed microbiome.” Phytobiomes Journal 3(2), 148–157. <https://doi.org/10.1094/PBIOMES-01-19-0009-R>
2. Bolyen, Evan, Rideout, Jai Ram, Dillon, Matthew R., Bokulich, Nicholas A., Abnet, Christian C., Al-Ghalith, Gabriel A., Alexander, Harriet, Alm, Eric J., Arumugam, Manimozhiyan, Asnicar, Francesco, Bai, Yang, Bisanz, Jordan E., Bittinger, Kyle, Brejnrod, Asker, Brislawn, Colin J., Brown, Christopher T., Callahan, Benjamin J., Caraballo-Rodríguez, Andrés M., Chase, Jason, Cope, Emily K., Da Silva, Raquel, Diener, Christian, Dorrestein, Pieter C., Douglas, Gregory M., Durall, Daniel M., Duvallet, Claire, Edwardson, Christian F., Ernst, Madeleine, Estaki, Mehrbod, Fouquier, Jennifer, Gauglitz, Julia M., Gibbons, Sean M., Gibson, Deanna L., Gonzalez, Antonio, Gorlick, Katherine, Guo, Jun, Hillmann, Benjamin, Holmes, Susan, Holste, Hannah, Huttenhower, Curtis, Huttley, Gavin A., Janssen, Stefan, Jarmusch, Alan K., Jiang, Lin, Kaehler, Benjamin D., Kang, Kyung B., Keefe, Christopher R., Keim, Paul, Kelley, Scott T., Knights, Dan, Koester, Ingrid, Kosciolek, Tomasz, Kreps, Jason, Langille, Morgan G. I., Lee, Jason, Ley, Ruth, Liu, Yu-Xing, Loftfield, Emily, Lozupone, Catherine, Maher, Michael, Marotz, Clarisse, Martin, Benjamin D., McDonald, Daniel, McIver, Lauren J., Melnik, Alexey V., Metcalf, Jessica L., Morgan, Shannon C., Morton, James T., Naimey, Aaron T., Navas-Molina, Jose A., Nothias, Louis-Félix, Orchanian, Sarah B., Pearson, Todd, Peoples, Samuel L., Petras, Daniel, Preuss, Matthew L., Pruesse, Elmar, Rasmussen, Lars B., Rivers, Aaron, Robeson II, Michael S., Rosenthal, Peter, Segata, Nicola, Shaffer, Michael, Shiffer, Adam, Sinha, Rashmi, Song, Se Jin, Spear, John R., Swafford, Austin D., Thompson, Luke R., Torres, Paul J., Trinh, Phuong, Tripathi, Anupriya, Turnbaugh, Peter J., Ul-Hasan, Shahjahan, van der Hooft, Justin J. J., Vargas, Florencia, Vázquez-Baeza, Yoshiki, Vogtmann, Emily, von Hippel, Matthew, Walters, William, Wan, Yanyan, Wang, Mingxun, Warren, Jessica, Weber, Kelly C., Williamson, Charles H. D., Willis, Amy D., Xu, Zhenjiang Zech, Zaneveld, Jesse R., Zhang, Yanjun, Zhu, Qiyun, Knight, Rob, and Caporaso, J. Gregory. (2019). “Reproducible, interactive, scalable and extensible microbiome data science using QIIME 2.” *Nature Biotechnology*, 37(8), 852–857. <https://doi.org/10.1038/s41587-019-0209-9>
3. R Core Team. 2022. “R: A language and environment for statistical computing.” R Foundation for Statistical Computing, Vienna, Austria. <https://www.r-project.org/>
4. Nygaard, Vegard, Einar Andreas Rødland, and Eivind Hovig. 2016. “Methods that remove batch effects while retaining group differences may lead to exaggerated confidence in downstream analyses.” *Biostatistics* 17 (1): 29–39. <https://doi.org/10.1093/biostatistics/kxv027>
5. Sims, Andrew H., Smethurst, Graeme. J., Hey, Yvonne., Okoniewski, Michal. J., Pepper, Stuart. D., Howell, Anthony., Miller, Crispin. J., and Clarke, Robert. B. 2008. “The removal of multiplicative, systematic bias allows integration of breast cancer gene expression datasets – improving meta-analysis and prediction of prognosis.” BMC Medical Genomics 1(1), 1–14. <https://doi.org/10.1186/1755-8794-1-42>
6. Hornung, Roman, Boulesteix, Anne-Laure, and Causeur, David. 2016. “Combining location-and-scale batch effect adjustment with data cleaning by latent factor adjustment.” BMC Bioinformatics 17(1), 1–19. <https://doi.org/10.1186/s12859-015-0870-z>
7. Zhou, Longjian., Sue, Andrew. C.-H., and Goh, Wilson. W. B. 2019. “Examining the practical limits of batch effect-correction algorithms: When should you care about batch effects?” Journal of Genetics and Genomics 46(9), 433–443. <https://doi.org/10.1016/j.jgg.2019.08.002>
8. Wang, Yiwen, and Lê Cao, Kim-Anh. 2023. “PLSDA-batch: a multivariate framework to correct for batch effects in microbiome data.” Briefings in Bioinformatics, 1–17. <https://doi.org/10.1093/bib/bbac622>
9. Ling, Wodan., and Wu, Michael. 2022. “ConQuR: batch effects removal for microbiome data in large-scale epidemiology studies via conditional quantile regression.” R Package Version 1.0.
10. Prado, Alberto, Marolleau, Brice., Vaissière, Bernard. E., Barret, Matthieu., and Torres-Cortes, Gloria. 2020. “Insect pollination: an ecological process involved in the assembly of the seed microbiota.” Scientific Reports 10(1), 1–11. <https://doi.org/10.1038/s41598-020-60591-5>
11. Reese, Sarah E. 2013. “Detecting and correcting batch effects in high-throughput genomic experiments.” Thesis, *Virginia Commonwealth University*: 1–136.
12. Ewels, Phil. 2021. “SRA Explorer.” https://sra-explorer.info/#
13. Callahan, Benjamin. J., McMurdie, Paul. J., Rosen, Michael. J., Han, Andrew. W., Johnson, Amy Jo. A., and Holmes, Susan. P. 2016. “DADA2: High-resolution sample inference from Illumina amplicon data.” Nature Methods 13(7), 581–583. <https://doi.org/10.1038/nmeth.3869>
14. Rideout, Jai R., Chase, John. H., Bolyen, Evan., Ackermann, Gail., González, Antonio., Knight, Rob., and Caporaso, J. Gregory. 2016. “Keemei: cloud-based validation of tabular bioinformatics file formats in Google Sheets.” GigaScience 5(27). <https://doi.org/10.1186/s13742-016-0133-6>
15. McMurdie, Paul J., and Holmes, Susan. 2013. “phyloseq: An R Package for Reproducible Interactive Analysis and Graphics of Microbiome Census Data.” PLoS ONE 8(4). <https://doi.org/10.1371/journal.pone.0061217>
16. van den Boogaart, K. Gerald, Tolosana-Delgado, Raimon., and Bren, Matevz. 2022. “compositions: Compositional Data Analysis.” CRAN, v2.0-4.
17. Lubbe, Sugnet, Filzmoser, Peter, and Templ, Matthias. 2021. “Comparison of zero replacement strategies for compositional data with large numbers of zeros.” Chemometrics and Intelligent Laboratory Systems 210(January), 104248. <https://doi.org/10.1016/j.chemolab.2021.104248>
18. Gloor, Gregory B., Macklaim, Jean M., Pawlowsky-Glahn, Vera, and Egozcue, Juan. J. 2017. “Microbiome datasets are compositional: And this is not optional.” Frontiers in Microbiology 8(NOV), 1–6. <https://doi.org/10.3389/fmicb.2017.02224>
19. Foxx, Alicia J., and Rivers, Adam. R. 2023. “Data: Benchmarking batch correction methods for synthesizing imbalanced microbiome community profiles.” Mendeley Data. <https://doi.org/10.17632/5xrfg5dym6.1>
